# Dr. Merriman's Correction of a Verbal Error

**Published:** 1813-04

**Authors:** Samuel Merriman

**Affiliations:** Half-moon-street


*To the Editors of the Medical and Physical Journal.*

GENTLEMEN,

I BEG leave, by means of your Journal, to correct a verbal error which I have unintentionally committed, in a Paper on "Premature Labor artificially induced in Women with distorted Pelves," lately published in the third volume of the "Medico-Chirurgical Transactions." The subject of my paper leading me to refer to a communication on the same subject, by Mr. *John* Barlow, of Bolton, in Lancashire, published in the eighth volume of "Medical Facts and Observations," I inadvertently wrote the word "*Blackburn*"

instead of "*Bolton*," and thus appear to attribute to Mr. *James* Barlow, of *Blackburn*, what was really written by Mr. *John* Barlow, of *Bolton*.

I am the more desirous of correcting this error, as soon as possible, because Mr. *James* Barlow has likewise published on this subject, in the fifth and ninth volumes of your Journal; and his opinions do not in every respect coincide with those of Mr. *John* Barlow.

Since my paper in the "Medico-Chirurgical Transactions" was published, I have learnt that Mr. Barlow, of Bolton, is not now living; he would therefore have been more correctly designated as *the late Mr. Barlow*.

*Half-moon-street*, I remain, &c.

*March* 10, 1813. SAMUEL MERRIMAN.

